# 2-Hydroxyglutarate Metabolism Is Altered in an *in vivo* Model of LPS Induced Endotoxemia

**DOI:** 10.3389/fphys.2020.00147

**Published:** 2020-03-03

**Authors:** Susan F. Fitzpatrick, Simon Lambden, David Macias, Zudin Puthucheary, Sandra Pietsch, Lee Mendil, Mark J. W. McPhail, Randall S. Johnson

**Affiliations:** ^1^Department of Physiology, Development and Neuroscience, University of Cambridge, Cambridge, United Kingdom; ^2^Department of Anesthesia and Intensive Care, Royal Free London NHS Foundation Trust, Centre for Health and Human Performance, University College London, London, United Kingdom; ^3^Centre for Human, Aerospace and Physiological Sciences, King’s College London, London, United Kingdom; ^4^CRUK, Addenbrooke’s Hospital, University of Cambridge, Cambridge, United Kingdom; ^5^Department of Inflammation Biology, Institute of Liver Studies, King’s College London, London, United Kingdom

**Keywords:** 2-hydroxyglutarate, endotoxemia, sepsis, hypothermia, 2-hydroxygluterate dehydrogenase, iNOS

## Abstract

The metabolic response to endotoxemia closely mimics those seen in sepsis. Here, we show that the urinary excretion of the metabolite 2-hydroxyglutarate (2HG) is dramatically suppressed following lipopolysaccharide (LPS) administration *in vivo*, and in human septic patients. We further show that enhanced activation of the enzymes responsible for 2-HG degradation, D- and L-2-HGDH, underlie this effect. To determine the role of supplementation with 2HG, we carried out co-administration of LPS and 2HG. This co-administration in mice modulates a number of aspects of physiological responses to LPS, and in particular, protects against LPS-induced hypothermia. Our results identify a novel role for 2HG in endotoxemia pathophysiology, and suggest that this metabolite may be a critical diagnostic and therapeutic target for sepsis.

## Introduction

Sepsis is defined as the dysregulated host response to infection (Singer et al., 2016) and is a major cause of morbidity and mortality worldwide. The number of cases seen continues to increase, in part due to a rise in the number of pathogens with antimicrobial resistance (AMR). However, therapeutic and diagnostic options remain limited. Therefore, there is an urgent need to understand its pathophysiology to develop novel diagnostic and therapeutic strategies.

In a healthy individual metabolism is characterized by synchronized anabolic and catabolic processes which act to maintain cell homeostasis. However, in septic patients, intense metabolic dysfunction occurs; contributing to sepsis pathophysiology and its sequelae of organ failure (Chioléro et al., 1997). In recent years, metabolic profiling has been explored as a novel means to approach diagnosis, prognosis and monitoring of septic patients (Seymour et al., 2013; Su et al., 2014; Nguyen et al., 2015). Administration of the bacterial endotoxin, lipopolysaccharide (LPS, the outer component of gram-negative bacteria), replicates the physiology and metabolic changes seen in sepsis (Kamisoglu et al., 2015). Indeed, a previous study showed concordance in plasma metabolomes between LPS-treated subjects and septic patients (Kamisoglu et al., 2015). Thus the endotoxemia model is a relevant physiological model of sepsis, which also provides important insights into the metabolic changes associated with sepsis.

2-Hydroxyglutarate (2HG), a key metabolite of the butanoate metabolic pathway, is produced by phosphoglycerate dehydrogenase catalyzing the NADH-dependent reduction of α-ketoglutarate (αKG) to 2HG (Nadtochiy et al., 2016). In humans two enantiomers exist, R- and S-2HG, which are normal endogenous metabolites present in all body fluids. The R-enantiomer has also been shown to be generated by gain-of-function mutations in the isocitrate dehydrogenase isoforms, which are associated with numerous types of cancer (Dang et al., 2009; Gross et al., 2010; Komotar et al., 2010). The S-enantiomer has been demonstrated to be produced under hypoxic conditions by lactate dehydrogenase (Intlekofer et al., 2015) and malate dehydrogenase (Oldham et al., 2015) and by lactate dehydrogenase in the testis (Teng et al., 2016). In addition to its regulation by synthesis, the enzymes L- or D-2HG dehydrogenase (L- or D-2HGDG) normally keep the level of 2HG low by promoting its conversion back to αKG (Nadtochiy et al., 2016). Mutations in these enzymes produce the inherited metabolic disease hydroxyglutaric acidosis (Nadtochiy et al., 2016). More recently, the physiological role of 2HG has begun to be elucidated. S-2HG has been shown to play a critical role as an immunometabolite in shaping the T-cell response (Tyrakis et al., 2016), while R-2HG has also been implicated in shaping the immune response (Böttcher et al., 2018). Thus, 2HG may have an important role in inflammatory and immunological pathophysiology.

To date, the majority of studies have focused on the tumor promoting roles of 2HG, but its contribution to the pathophysiology of other disease states, including sepsis, remains to be elucidated. In this study, using both murine models of LPS-induced endotoxemia, and samples from human septic patients, we investigated the changes in 2HG metabolism. We subsequently determined the therapeutic potential of 2HG and the mechanism underlying our observations.

## Materials and Methods

### Wild-Type Mices

All procedures involving mice were approved by the University of Cambridge, Animal Ethics Committee. Wild type male mice in a C57/Bl6 background were purchased from Charles Rivers. fr. Mice between 8 and 12 weeks were used for experiments. Urine was obtained prior to the start of the experiments. Mice were then intraperitoneally (i.p) administered 15 mg/kg of ultrapure LPS (Fitzpatrick et al., 2018) (InvivoGen) and/or 50 mg/kg of S-2HG (Tyrakis et al., 2016), IL-4 (20 mg/kg) or IFNgamma (20 mg/kg) (R&D, United Kingdom). Mice were culled at the end of the experimental protocol. Urine was collected at various times post i.p. Urine was collected without intervention as previously outlined (Kurien et al., 2004).

#### Transgenic Mice

HIF-1DF/LysM-Cre male transgenic mice in C57BL/6 background were generated as previously described (Takeda et al., 2010). Cre-negative homozygous male littermates for the conditional alleles were used as controls. Animals were between 8 and 10 weeks at the time of experiments. Urine was obtained prior to the start of the experiment and mice were subsequently administered LPS (15 mg/kg) i.p.

### Cell Culture

Bone marrow derived macrophages (BMDM) were isolated from the femurs and tibias obtained from male C57/Bl6 wild type mice (8–10 weeks old) as previously described (Takeda et al., 2010). Briefly, cells were plated in DMEM supplemented with 10% heat inactivated FBS and 30% conditioned media (supernatant from M-CSF expressing L929 cells). Adherent BMDM were harvasted using 0.05% Trypsin EDTA after 7 days in culture. Ultrapure LPS was obtained from Invitrogen and R/S 2HG Octyl Ester Sodium salt were obtained from Toronto Research Canada.

### Human Critical Illness Samples

This study was undertaken in a group of patients that had previously been included in an observational study of the impact of critical illness on muscle function (16). Patients were those enrolled in the previously described MUSCLE study (NCT01106300^1^). Ethical approval was obtained from University College London Ethics Committee A. At enrollment, written assent was obtained from the next-of-kin with retrospective patient consent obtained when full mental capacity was regained. During this study, urine samples were collected at inclusion (ICU admission) from urinary catheters and on study days 3, 7, and 10 in patients expected to spend longer than 3 days in the ICU. Urine samples were stored in aliquots at −80°C until analysis.

### R-2HG Colorimetric Assay

Media was obtained from BMDM’s treated with LPS for 4 h. Urine was obtained from mice before and at various times after LPS (i.p) and serum was obtained from septic patients. All samples were frozen at -80.R-2HG levels were quantified using a R-HG Colorimetric kit (Biovision, California, United States) according to the manufacturer’s guidelines.

### 2-HG Mass Spectroscopy

Extraction of 2HG from lysed cell pellets and urine samples was performed using methanol protein precipitation (containing deuterated 2HG internal standard) followed by derivatization of the enantiomers using Diacetyl-L-tartaric anhydride (DATAN). LC-MS/MS analysis was performed using a Shimadzu Nexera X2 UHPLC system coupled to a Sciex Triple Quad 6500 Mass Spectrometer. Derivatized enantiomers of 2HG and internal standard were separated on a Waters Acquity UPLC HSS T3, 100 × 2.1 mm, 1.8 μm column using a 125 mg/L Ammonium Formate (aq) pH3.6/Acetonitrile gradient.

### Radiotelemetry Measurements

Radio-transmitters (DSI, United States) were surgically implanted into wild type mice weighing >25 g, under anesthesia as previously described (Fitzpatrick et al., 2018). 10 days post-surgery the transmitters were turned on and baseline temperature, blood pressure and heart rate recordings were obtained. LPS (15 mg/kg) and 2HG (50 mg/kg) i.p were administered under anesthesia. Once mice had recovered from the anesthetic, additional recordings were obtained for a further 6 h.

### Real-Time PCR (RT-PCR)

Total RNA was isolated using an UltaClean Tissue and Cell RNA isolation kit (Mobio, Canada). cDNA was synthesized from 1 ug of total RNA using Superscript III (Invitrogen, United Kingdom) according to the manufacturers protocol. Relative abundance of transcripts were assessed by Q-PCR following normalization to B2M or 18S. QuantiTect Primer Assays (Qiagen, United Kingdom) were obtained for all transcripts.

### Immunoblotting

Protein was isolated using RIPA buffer and normalized using a Bradford assay. Ten micrograms of whole cell protein were separated on 4–8% Tris-acetate gels (Invitrogen, United Kingdom) followed by immunoblotting. Primary antibodies for L2HGDG, D2HGDG, Bactin (Thermo Fisher, United Kingdom), HIF-1α and iNOS (Novus, United Kingdom) were subsequently probed with secondary antibodies from Amersham and visualized using ECL plus (GE Healthcare, United Kingdom).

### Arginase Activity Assay

Following treatment BMDM were lysed in an arginase lyses buffer as previously described (Puthucheary et al., 2013). 50 uL of lysate was incubated with 75 uL of Tris–HCl (50 mmol/L, pH 7.5) containing 10 mmol/L MnCl2. The lysate was heated to 55°C for 10 min to activate the arginase. The hydrolysis reaction of L-arginine by arginase was performed by incubating the mixture containing activated arginase with 50 uL of L-arginine (0.5 mol/L, pH 9.7) at 37°C for 1 h. The reaction was subsequently stopped by adding 400 uL of the acid solution mixture (H2SO4:H3PO4:H2O1:3:7). For colorimetric determination of urea, -isonitrosopropiophenone (25 uL, 9% in absolute ethanol) was added and the mixture was incubated at 100°C for 45 min. After placing the sample in the dark for 10 min at room temperature, the urea concentration was determined spectrophotometrically by the absorbance at 550 nm measured with a microplate reader (BioTek Instruments). The amount of urea produced, after normalization with protein, was used as an index for arginase activity.

### Extracellular Flux Analysis

Bone marrow derived macrophages were seeded at a density of 70,000 cells per well 24 h prior to the assay. Following a media change cells were left untreated or treated with LPS alone, S-2HG or LPS + S-2HG for 4 h. OCR were measured on an XFe24 Analyzer (Seahorse Bioscience). The mitochondrial stress test was performed according to the manufacturer’s protocol.

### Statistical Analysis

All statistical analysis was performed using Prism (Graphpad software). If not otherwise stated, a two-sided unpaired Students *t*-test or ANOVA was used to analyze the differences in means between the treatment groups.

## Results

### Urinary Excretion of 2HG Is Suppressed in Sepsis

**FIGURE 1:**
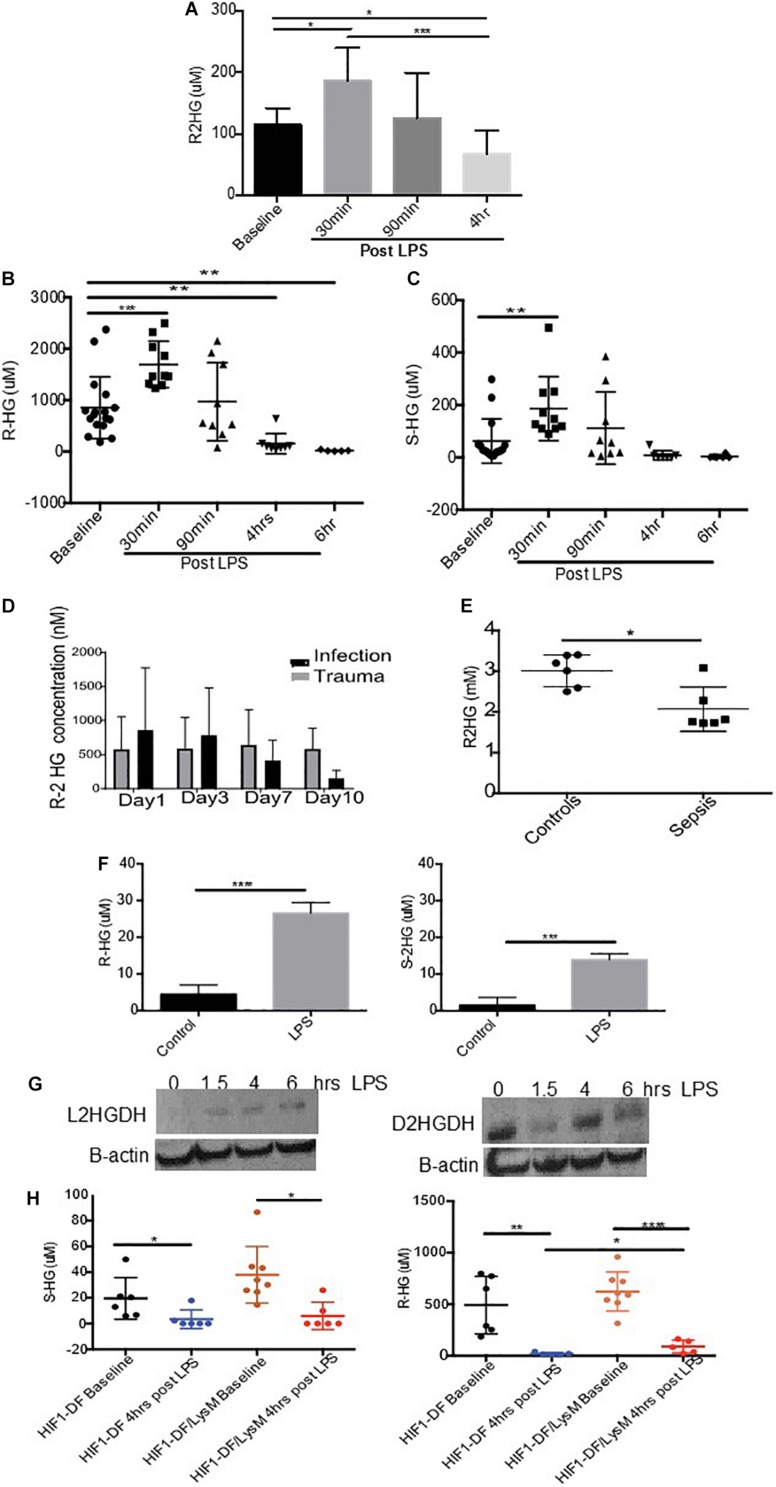
Urinary excretion of 2-HG is suppressed in sepsis. Mice were treated with LPS (15 mg/kg) and urine was obtained over a time course 0–4 h. Urine samples were subsequently measured using a **(A)** colorimetric assay for R-2HG or mass spectroscopy for **(B)** R- and **(C)** S-2HG. *N* ≥ 7 for all time points. R-2HG levels was quantified in **(D)** urine or **(E)** serum isolated from septic patients. **(F)** R- and S-2HG were quantified by mass spectroscopy in BMDM treated with LPS (1 ug/ml) **(G)** BMDM were treated with LPS (1 ug/ml). Immunoblotting for L/D-2HGDG protein was performed and quantified by densitometry analysis. *N* = 3. **(H)** Urine was taken from wild type (HIF-1DF) and HIF-1DF/LysM mice at baseline. Following LPS (15 mg/kg) administration urine was obtained after 4 h and mass spectroscopy for both R- and S-2HG was performed. *N* = 6–8 for all time points. * *p* < 0.05,. ** *p* < 0.01, *** *p* < 0.005, and **** *p* < 0.001.

We began by investigating the impact of LPS stimulation on the urinary excretion of 2-HG, in wild type C57/Bl6 mice. Urine was obtained from mice prior to and at various time points post LPS (15 mg/kg I.p.) administration. An initial increase in R-2HG excretion after 30 min (*p* < 0.05), was followed by a significant suppression in urinary excretion by 4 h (*p* < 0.05) (Figure 1A). We subsequently utilized mass spectroscopy to quantify both R- and S-2HG urinary concentrations. In agreement, with the previous experiment a significant elevation in urinary R-2HG excretion occurred after 30 min and loss of excretion by 4 h (Figure 1B). Furthermore, a similar effect was also observed on S-2HG excretion (Figure 1C). However, neither R- or S-2HG urinary excretion was significantly altered by the administration of the cytokines IL- 4 (Supplementary Figures 1) or IFNgamma (Supplementary Figures 2). Administration of LPS *in vivo* replicates the physiology of sepsis. Thus, we determined the effect of sepsis on urinary R-2HG excretion in ICU patients. A colorimetric assay for R-2HG, showed that R-2HG excretion could be used to distinguish patients with sepsis from those with trauma (Figure 1D). Furthermore, serum levels of R-2HG was reduced in serum isolated from septic patients (Figure 1E). LPS induces significant changes in innate immune cells. We next investigate the effect of LPS stimulation on BMDM. A significant increase in both R- and S-2HG was observed following LPS stimulation (Figure 1F).

### LPS Promotes 2HG Degradation Through a HIF-1/2HGDG Pathway

We subsequently investigated the mechanism responsible for reduced urinary excretion of 2HG. 2HGDG is a mitochondrial enzyme responsible for the conversion of 2HG to α ketoglutarate (αKG). To assess whether LPS altered 2HGDG activation, protein was obtained from BMDM treated with LPS. A significant increase in the protein expression of L-2HGDH was observed 4 h after LPS stimulation, while D-2HGDH was found to be initially suppressed and increased at 4 h (Figure 1G). HIF-1α has been shown to modulate 2HGDH and thus 2HG. In urine taken from HIF-1DF/LysM mice a significant protective effect on R-2HG but not S-2HG excretion was observed in comparison to the HIF-1DF controls following 6 h of LPS treatment (Figure 1H). These, results propose that the LPS induced activation of 2HGDG promotes the breakdown of 2HG, which may in part account for its reduced urinary excretion.

### 2-HG Protects Against LPS Induced Hypothermia

**FIGURE 2:**
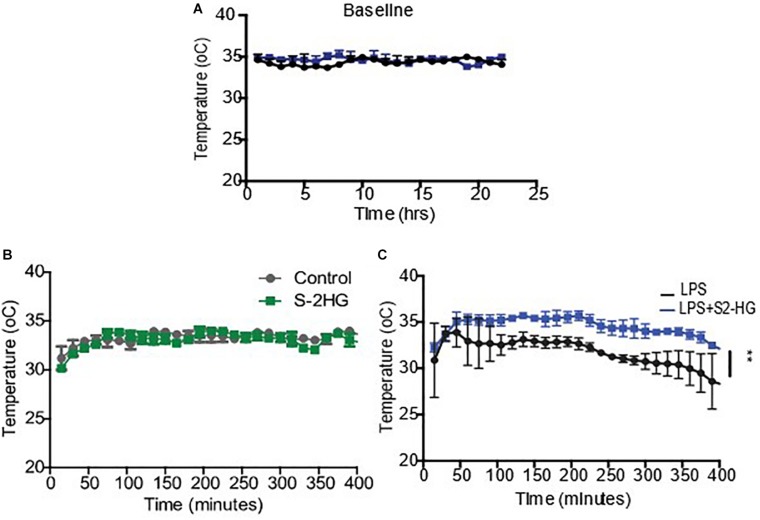
2-HG protects against LPS induced hypothermia. Mice were surgically implanted with a radiotelemetry probe. 10 days post-surgery **(A)** Baseline temperature recordings were obtained. Mice were subsequently treated **(B)** S-2-HG (50 ug/kg) **(C)** LPS (15 mg/kg) or a combination of LPS and S-2HG and temperature recordings were obtained for an additional 8 h. *N* = 4/5. * *p* < 0.05.

To determine if 2HG could have a therapeutic effect on LPS pathophysiology, we next investigated the impact of 2HG administration on LPS-induced symptomology *in vivo.* LPS-induced endotoxemia manifests as disruptions in heart rate, blood pressure and body temperature. Wild-type mice were surgically implanted with radiotelemetry probes. Ten days post-surgery baseline temperature, heartrate and blood pressure measurements were obtained for all treatment groups (Figure 2A and Supplementary Figures 3). 2HG administration did not affect LPS-induced tachycardia or hypotension (Supplementary Figures 3). Interestingly, while S-2HG alone had no effect on temperature (Figure 2B) co-administration of LPS and S-2HG was found to have a significant protective effect against LPS-induced hypothermia (Figure 2C).

### 2HG Protects Against LPS Driven HIF-1α Stabilization

**FIGURE 3:**
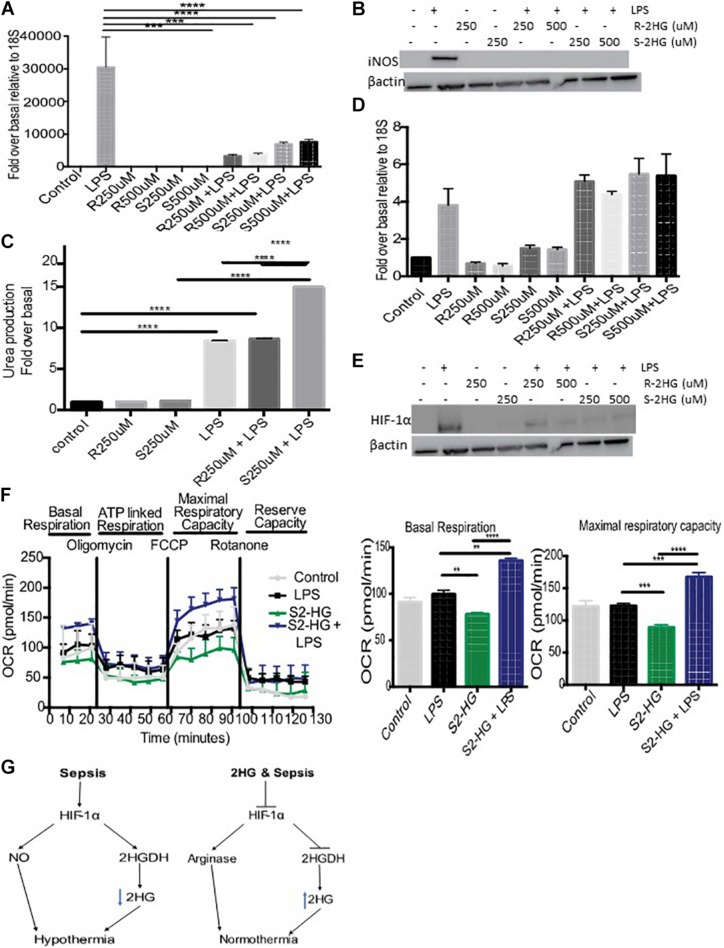
2HG protects against LPS driven HIF-1α stabilization. BMDB were treated with 250 or 500 uM R-/S-2HG in the presence or absence of LPS. 24 h post-stimulation **(A)** iNOS RNA expression was quantified by RT-PCR, **(B)** iNOS protein expression was determined by immunoblotting **(C)** Arginase activity was measured using a urea production assay. **(D)** HIF-1αmRNA was measured by RT-PCR **(E)** HIF-1α protein expression was measure by western blot analysis **(F)** BMDM were treat with LPS for 4 h and a Seahorse Mitochondrial Stress Test was carried out. *N* = 3–5 for all experiments. * *p* < 0.05, *** *p* < 0.005, **** *p* < 0.001.**(G)** Schematic of the role of 2HG in endotoxemia induced hypothermia.

Inducible nitric oxide synthase (iNOS) plays an important role in regulating hypothermia. Therefore, we investigated the degree to which S-2HG mediated its protective effects on LPS-induced hypothermia by regulating iNOS signaling. We observed enhanced iNOS mRNA and protein levels in LPS-treated BMDM. However, combined treatment with R- or S-2HG and LPS caused a significant suppression of both iNOS mRNA (*p* < 0.001) (Figure 3A) and protein expression (Figure 3B). Furthermore, a significant increase in arginase activity was observed in cells treated with S-2HG and LPS in comparison to LPS alone (*p* < 0.0001) (Figure 3C). HIF-1α is a key regulator of iNOS, in response to LPS (Takeda et al., 2010). Thus, we subsequently investigated the effect of 2HG on HIF-1α expression. LPS-stimulated up-regulation of HIF-1α mRNA expression was unaffected by the presence of either R- or S-2HG (Figure 3D). In contrast, the LPS-induced upregulation of HIF-1α protein expression was suppressed when cells were co-treated with LPS and R- or S-2HG (Figure 3E).

### S-2HG Improves Mitochondrial Respiration

Excessive NO production during sepsis results in mitochondrial dysfunction. We next tested the hypothesis that S-2HG protects against this. BMDM treated with a combination of S-2HG and LPS had a significant improvement in both basal respiration and maximal respiratory capacity in comparison to LPS or S-2HG alone (Figure 3F).

## Discussion

The response to endotoxemia is closely associated with alterations in metabolism, which closely mimic those seen in sepsis (Kamisoglu et al., 2015). Using a mouse model of LPS-induced endotoxemia, we showed that the temporal dynamics of the urinary excretion of the metabolite 2HG is significantly altered in response to endotoxemia. This is consistent with previous studies, which have shown that the dynamics of metabolic pathways and metabolites (e.g. glucose, amino acids, and lipids) are significantly altered in response to LPS and sepsis (Kamisoglu et al., 2013, 2015; Terenina et al., 2017; Fitzpatrick et al., 2018). Moreover, in septic patients 2HG levels were also reduced in both urine and serum samples. Recently, the use of urinary 2HG as a biomarker has been proposed for the identification of IDH-mutant gliomas and to predict an ulcerative colitis patients risk of developing colon cancer (Han et al., 2018). While further clinical studies are required our results suggest 2HG may have a potential role as a predictive biomarker for sepsis.

The molecular mechanisms governing the regulation of 2HG under physiological and pathophysiological conditions remain poorly understood. 2HG is produced from αKG (Nadtochiy et al., 2016), glutamine (Oldham et al., 2015), glucose (Teng et al., 2016), lactate dehydrogenase (Intlekofer et al., 2015; Teng et al., 2016), and malate dehydrogenase (Oldham et al., 2015). Endotoxemia and sepsis are associated with profound changes in numerous metabolic pathways including glycolytic, tricylic-acid cycle (TCA), glutamine and arginine metabolism (reviewed Fitzpatrick, 2019). Thus we hypothesis that the changes in the temporal dynamics of 2HG are a consequence of metabolic dysregulation. Consistent with this idea, in hypoxia TCA dysfunction leads to L-2HG accumulation (Oldham et al., 2015). Point mutations in the 2HGDH enzymes also lead to the accumulation of 2HG and studies in drosophila, have reported that metabolic products are key regulators of L2HGDH (Li et al., 2018). We reasoned that endotoxemia induced changes in 2HG maybe a consequence of altered 2HGDH levels. Suppressed 2-HG excretion correlated with an increase in the expression of 2HGDG. It has recently been shown that HIF-1α expression is directly proportional to D2HGDH, and that HIF-1α can modulate 2HG levels by binding directly to the D2HGDH promoter (Han et al., 2018). Consistent, with these studies we observed increased urinary 2HG levels following loss of HIF-1α. We propose that 2HG is suppressed in part due to the activation of a HIF-1α/2HGDH signaling pathway (Figure 3G).

The enantiomers of 2HG serve as important regulators of cellular redox homeostasis, cellular energy metabolism and the immune system (Oldham et al., 2015; Böttcher et al., 2018). Consequently, temporal changes in 2HG, in septic patients, will likely have a significant impact upon these processes, which in turn will contribute to clinical symptomology. We observed that S-2HG administration had a significant protective effect against LPS induced hypothermia. The etiology of hypothermia during sepsis is poorly understood. However, it has been shown to be related to mitochondrial dysfunction due to excessive nitric oxide production from iNOS (Saia and Carnio, 2006; Hiller et al., 2014). Our results demonstrated that S-2HG protects against NO generation. We believe that this is due in part to a protective effect of S-2HG on mitochondria function and an increase in mitochondrial antioxidant capacity. Consistent with this idea, studies have demonstrated that S-2HG application increases mitochondrial respiration (Böttcher et al., 2018), while in hypoxia R-2HG accumulation regulates cellular redox homeostasis by controlling bioenergetic pathways (Oldham et al., 2015). Furthermore, in LPS stimulated BMDM treated with S-2HG we observed a significant improvement in mitochondrial function.

An increasing number of studies have identified that mitochondrial dysfunction contributes to epigenetic modification (reviewed Minocherhomji et al., 2012). Thus, given the literature showing 2HG as an epigenetic modifier, it is reasonable to predict that S-2HG may cause epigenetic modification of the iNOS promoter, leading to its suppressed activity (Rigillo et al., 2018). Inhibition of 2-oxoglutarate-dependent dioxygenases, that demethylate histones (Jumonji C containing proteins) or oxidize 5-methylcytosine in DNA (Ten-eleven translocation (Tet) proteins) could mediate S-2HG’s effect. Indeed, in CD8^+^ T cells S-2HG induces global methylation changes and small changes in 5-methylcytosine (Tyrakis et al., 2016).

In addition, to its effects at suppressing oxidative signaling, S-2HG could also promote anti-oxidative pathways. Nuclear factor-eythroid 2-related factor (Nrf2), a key transcription factor involved in activation of antioxidative and cytoprotective genes, is activated by DNA demethylation and histone methylation (Kang and Hyun, 2017). Furthermore, Nrf2 is also a key regulator of metabolism and mice with constitutive Nrf2 improved mitochondrial function, as demonstrated by higher OCR levels (Ohl et al., 2018). This is consistent with the increased OCR we observed in LPS-stimulated BMDM’s treated with S-2HG. Future studies will focus on understanding the interact between 2HG, redox homeostasis and epigenetics in sepsis models.

HIF-1α, the master regulator of the hypoxia response, plays a vital role in iNOS activation (Takeda et al., 2010). Moreover, previous studies have demonstrated that loss of HIF-1α signaling protects against endotoxemia and sepsis-induced hypothermia (Peyssonnaux et al., 2007; Mahabeleshwar et al., 2011; Fitzpatrick et al., 2018). Indeed, we observed HIF-1α protein destabilization upon S-2HG treatment. Earlier studies have shown that 2HG accumulation negatively impacts HIF-1α stability, an effect due in part to enhanced proteasomal degradation by the prolyl hydroxylase enzymes (Intlekofer et al., 2015). Thus, suppressed HIF-1α signaling could contribute to the decreased iNOS signaling and subsequent normothermia following S-2HG treatment (Figure 3G).

In summary, our results have identified a novel role for 2HG in endotoxemia and show that S-2HG protects against endotoxemia induced hypothermia. Cellular metabolism and oxidative stress are intimately linked. S-2HG through epigenetic modification may suppress oxidative signaling, while enhancing antioxidative signaling, which in turn could have a protective effect on endotoxemia-induced hypothermia. Determining the detailed signaling mechanism involved will identify novel therapeutic targets for sepsis treatment.

## Data Availability Statement

All datasets generated for this study are included in the article/Supplementary Material.

## Ethics Statement

All procedures involving mice were approved by the University of Cambridge, Animal Ethics Committee.

## Author Contributions

SF designed and performed the experiments and data analysis, and wrote the manuscript. SL and ZP isolated and analyzed the human patient data. DM performed the radiotelemetry implantation surgery. LM performed the mass spectroscopy experiments and analysis. SP aided in performing the human studies. MM supplied patient samples. RJ designed the experiments, wrote the manuscript, and administered the project.

## Conflict of Interest

The authors declare that the research was conducted in the absence of any commercial or financial relationships that could be construed as a potential conflict of interest.

## Footnotes

1 www.clinicaltrials.gov

## Supplementary Material

FIGURE S1 Mice were treated with IL-4 (20 mg/kg) and urine was obtained over a time course 0–4 h. Urine samples were subsequently measured by mass spectroscopy for both R- and S-2HG. *N* = 4 or more.

FIGURE S2 Mice were treated with IFNgamma (20 mg/kg) and urine was obtained over a time course 0–4 h. Urine samples were subsequently measured by mass spectroscopy for both R- and S-2HG. *N* = 4 or more.

FIGURE S3 Following radiotelemetry implantation baseline measurements were obtained for systolic and diastolic blood pressure, and heart rate. Mice were subsequently treated with LPS (15 mg/kg) or a combination of LPS and S-2HG (50 ug/kg) and recordings were obtained for an additional 8 h. *N* = 4/5.
