# Supplementary figures and images for: 2-Hydroxyglutarate Metabolism Is Altered in an in vivo Model of LPS Induced Endotoxemia
Source: Front Physiol. 2020 Mar 3;11:147. doi: 10.3389/fphys.2020.00147 (PMC7063103; doi:10.3389/fphys.2020.00147)

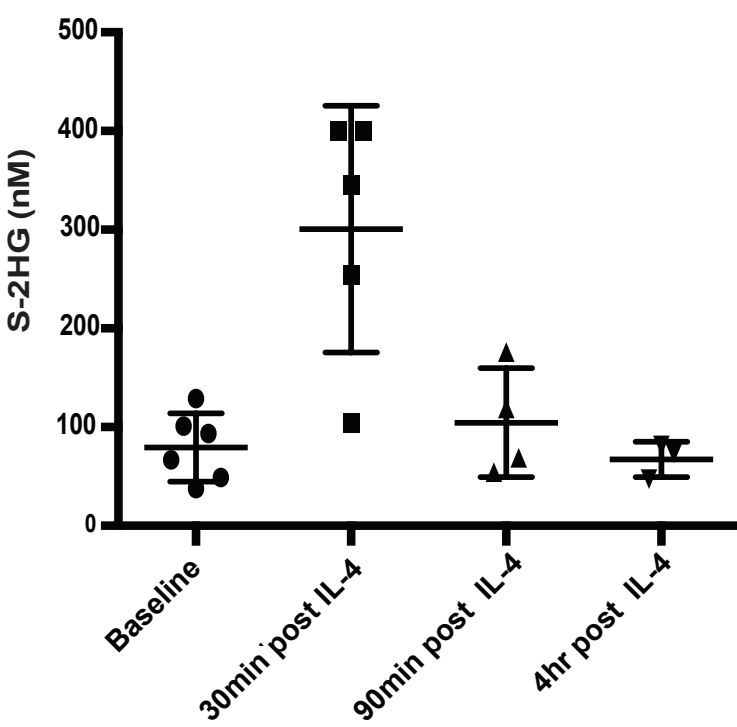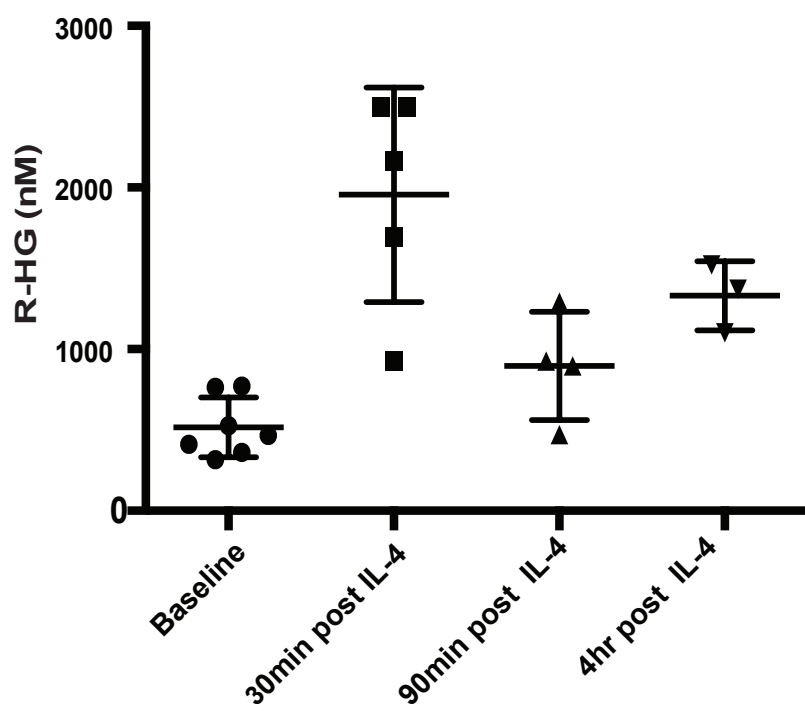

Supplementary Figure 1

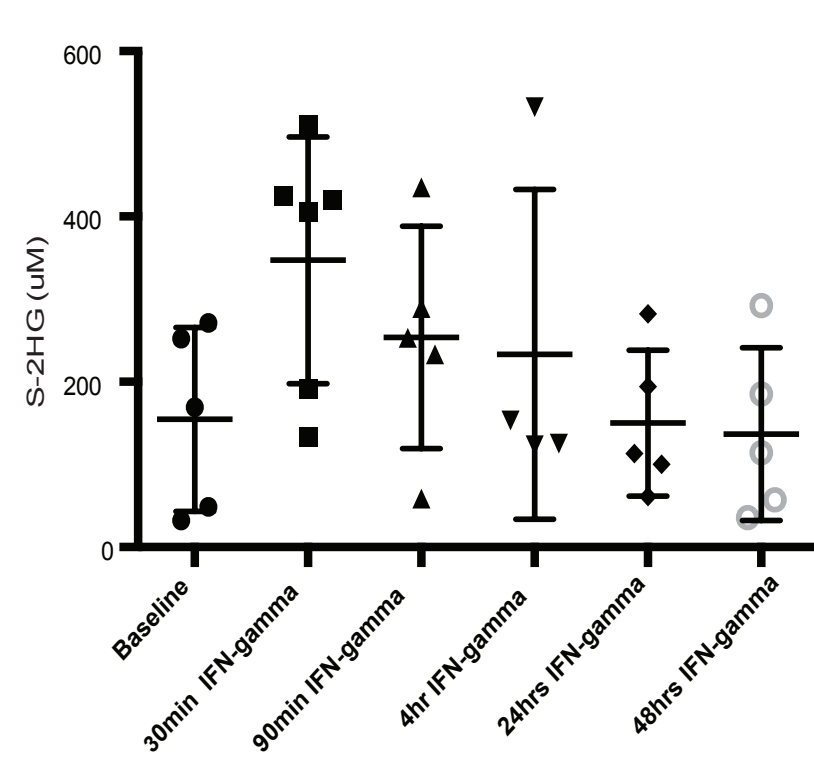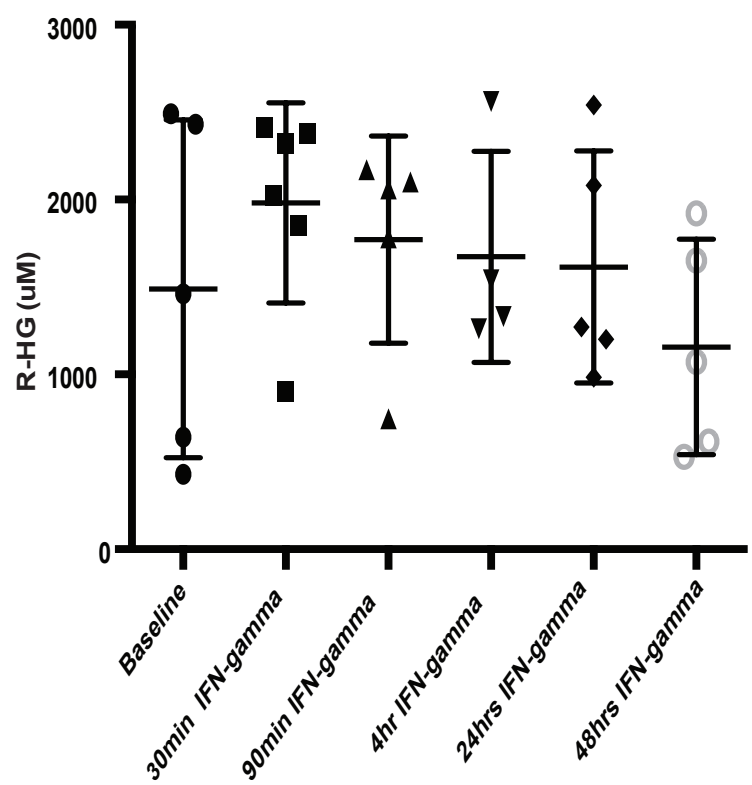

Supplementary Figure 2

## BASELINE

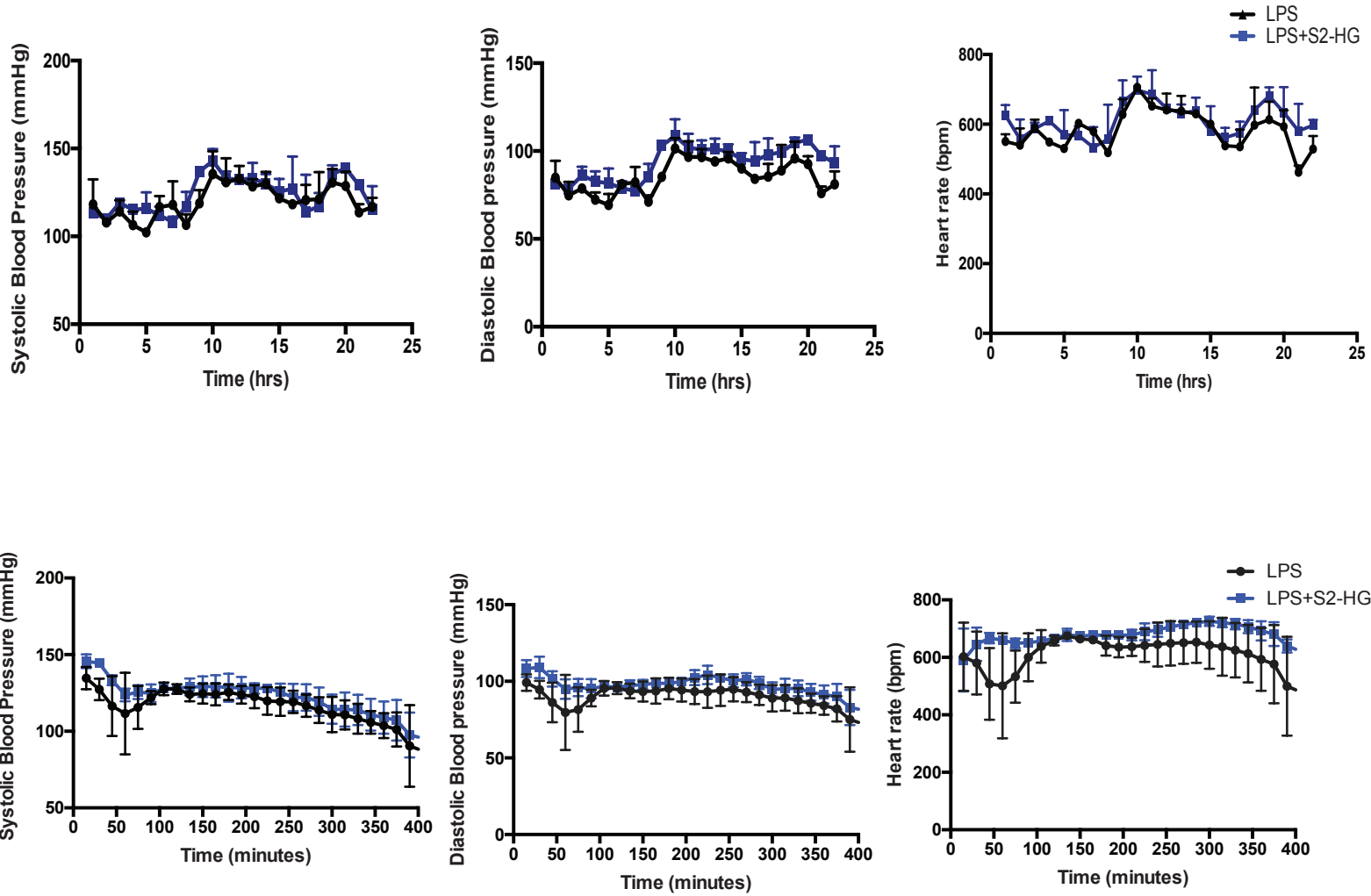

Supplementary Figure 3

Supplement: FIGURE S1 — Mice were treated with IL-4 (20 mg/kg) and urine was obtained over a time course 0–4 h. Urine samples were subsequently measured by mass spectroscopy for both R- and S-2HG. N = 4 or more. [file Data_Sheet_1.pdf]
